# Regulatory T cells from the inflamed joints of patients with juvenile idiopathic arthritis (JIA) are dysregulated and display a pro-inflammatory profile

**DOI:** 10.1186/1546-0096-9-S1-P288

**Published:** 2011-09-14

**Authors:** AM Pesenacker, Stavroula Zavitsanou, Kiran Nistala, Lucy R Wedderburn

**Affiliations:** 1 Rheumatology Unit, Institute of Child Health UCL, London, UK

## Background

Regulatory T cells (Treg) are abundant in the inflamed joints of children with JIA. However the high number of Treg is not able to control the inflammation within the joint, suggesting a shortcoming of their regulatory function. It has been suggested that Treg can develop a pro-inflammatory profile that is specifically related to the local pro-inflammatory response. In this study we investigated whether Treg within the inflamed joint of JIA patients express pro-inflammatory markers.

## Aim

This study examines the pro-inflammatory potential of Treg and how this might impact their regulatory function within the inflamed joint of children with JIA.

## Methods

Peripheral blood mononuclear cells (PBMC) and synovial fluid mononuclear cells (SFMC) of 12 children with JIA were analysed by flow cytometry for Treg, their maturation and lineage markers, and cytokine production.

## Results

In the inflamed joint of children with JIA an increase of cytokine producing Treg was observed compared to patient PBMC. A mean of 2.13% of all CD4 T cells were IFN-γ and FoxP3 positive in the SFMC compared to 0.30% in the PBMC of JIA patients (p<0.001). IL-17 expressing Treg were also increased in the joint compared to peripheral blood (p=0.01). Surprisingly a significantly higher proportion (1.34%, p=0.01) of all CD4+ synovial T cells co-expressed IL-2 and FoxP3. These cells conformed to other classical definition of Treg, including high expression of CD25 and low expression of CD127. Cytokine positive Treg were enriched within the CD161+ population, a known marker for pro-inflammatory Th17 T cell population.

## Conclusion

We demonstrate here that within the inflamed joint of children with JIA Treg are more likely to produce pro-inflammatory cytokines, than Treg from the blood. This may diminish regulatory function of Treg in the joint, and could in part explain the paradox of a high number of Treg in the joint, yet an inability to control disease. We identified CD161 as a marker on a subpopulation of Treg with potential for pro-inflammatory cytokine production. This study contributes to the understanding of the dysregulation of Treg within the joint of JIA and thus proposing an underlying mechanism of ongoing disease pathology of JIA. This might lead to refined targets for novel therapeutics.

